# Molecular and Cellular Mechanisms of Bone and Cartilage Diseases

**DOI:** 10.3390/biomedicines11092492

**Published:** 2023-09-08

**Authors:** M. Alaa Terkawi

**Affiliations:** Department of Orthopedic Surgery, Faculty of Medicine, Graduate School of Medicine, Hokkaido University, Kita-15, Nish-7, Kita-ku, Sapporo 060-8638, Japan; materkawi@med.hokudai.ac.jp; Tel.: +81-11-706-5935; Fax: +81-11-706-6054

Bones and cartilage, the two most important parts of the musculoskeletal system, provide mobility and maintain the body’s posture. Diseases that affect bone and cartilage, such as osteoporosis (OP), osteoarthritis (OA), rheumatoid arthritis (RA), and low back pain, have a significant impact on society since they limit the mobility and dexterity of the population. This can lead to early retirement from work and a reduced ability of people to participate in their societal duties. Despite the advances made in therapeutics, surgical intervention is the only option available in most cases of musculoskeletal diseases. Thus, there is an urgent need for conservative therapeutic alternatives to surgical intervention that are not currently available in most cases of elderly individuals. A better understanding of the pathophysiology of bone and cartilage in these diseases would lead to the discovery of new therapies. The current Special Issue, *Molecular and Cellular Mechanisms of Bone and Cartilage Diseases*, focuses on molecular and cellular mechanisms underlining bone and cartilage pathology and novel therapeutic approaches that are designed to target the above diseases.

The current issue addresses a number of important aspects of the pathophysiology of bone and cartilage in musculoskeletal disorders. This issue includes six peer-reviewed articles, including one review, four research articles, and one communication. The review article by Terkawi et al. [1] provides insight into the recent findings and discoveries highlighting the involvement of low-grade inflammation mediated by damage-associated molecular patterns (DAMPs) in the pathogenesis of osteoarthritis (OA). DAMPs are the major stimuli for initiating inflammatory cycles in the joint and for promoting chondrocyte catabolic responses leading to cartilage degeneration. The authors discuss links between risk factors for OA and the increased production of DMAPs and propose that modulating communication between synovial macrophages and chondrocytes via targeting DAMPs from ECM damage or necrotic cells represents an excellent approach for dampening low-grade inflammation and the OA process. Importantly, the study recommends a course of treatment for OA that includes routine, regular exercise, and healthy lifestyles, along with nutritional antioxidant and probiotic supplementations, to reduce systemic inflammation mediated by DAMPs.

In this Special Issue, the first research topic by Eiter et al. [2] reports on a comparison of the metabolic processes and mitochondrial function of OA and non-OA chondrocytes in the presence or absence of inflammatory mediators. Importantly, although glycolysis is similar in non-OA and OA chondrocytes, OA induces persistent metabolic alterations in chondrocytes with increased cellular mechanisms for protecting their mitochondrial functions. Non-OA chondrocytes appear to respond more strongly to inflammatory mediators, and their responses are characterized by a decrease in mitochondrial ATP production and an increase in non-mitochondrial oxygen consumption rates as well as NO production. These results shed light on a new clue supporting the concept that targeting mitochondrial pathways with small molecules that regulate/improve the vital functions of chondrocytes offers a promising therapeutic strategy for the treatment of OA. The second topic was a quasi-experimental pilot study reported by Maffei et al. [3], demonstrating the effects of a 6-month exercise program on bone turnover biomarkers, physical performance, and fear of falling, leading to vertebral fractures in osteoporotic women. Despite the small number of samples in this study, the findings provide evidence for the importance of an exercise program in enhancing functional performance and reducing the risk of falls in postmenopausal women with osteoporosis. This suggests that clinicians should include exercise as an additional option for the management of osteoporosis in postmenopausal women with vertebral fractures. Meesters et al. [4] also reported a positive correlation between successful bone healing, the concentrations of arginine and ornithine, and the expression of Nos2. The study includes seventeen patients with atrophic long bone nonunions treated by autologous bone grafting using the reamer-irrigator-aspirator procedure. The striking findings of this study are that the detection of arginine concentrations and Nos2 expression in the reamed bone marrow aspirate obtained from a site distant from the nonunion could be a useful approach for predicting the outcomes of long bone nonunion treatment. The next editor choice was a study by Bonifacio et al. [5] that provided solid evidence for the beneficial effects of hyaluronic acid and amino acids on in vitro maturation and activation of human osteoblasts, suggesting that the use of hyaluronic acid and amino acid solutions as therapeutic agents for promoting bone regeneration and nonunion healing has considerable merit. Furthermore, given the lack of knowledge concerning the mechanism of heterotopic ossification in response to traumatic brain injury, Kesavan et al. [6] report on the development of an animal model for traumatic brain injury augmentation of heterotopic ossification in response to local injury. In this model, a traumatic brain injury was induced by a weight-drop model in mice, and heterotopic ossification was then induced by local injury to the Achilles tendon or fibula in mice. As a result, there was a four-fold increase in the ectopic bone in the injured leg. Another important finding of this study is that the hypoxic state at the injury site in the soft tissues of traumatic brain injury mice provides a suitable environment for facilitating the accumulation and activation of pericytes to form endochondral bone.

Albeit interesting, while the articles that appear in this issue provide new knowledge regarding the molecular and cellular mechanisms underlining bone and cartilage biology, much research and effort are still needed to achieve a better understanding as a step toward the development of therapeutic agents.
